# Abundancy of polymorphic CGG repeats in the human genome suggest a broad involvement in neurological disease

**DOI:** 10.1038/s41598-021-82050-5

**Published:** 2021-01-28

**Authors:** Dale J. Annear, Geert Vandeweyer, Ellen Elinck, Alba Sanchis-Juan, Courtney E. French, Lucy Raymond, R. Frank Kooy

**Affiliations:** 1 Department of Medical Genetics, University of Antwerp, Antwerp, Belgium; 2 NIHR BioResource, Cambridge University Hospitals NHS Foundation Trust, Cambridge Biomedical Campus, Cambridge, CB2 0QQ UK; 3 Department of Haematology, NHS Blood and Transplant Centre, University of Cambridge, Cambridge, CB2 0PT UK; 4 Department of Paediatrics, University of Cambridge, Cambridge, CB2 0QQ UK; 5 Department of Medical Genetics, Cambridge Institute for Medical Research, University of Cambridge, Cambridge, CB2 0XY UK

**Keywords:** Medical genetics, Neurodevelopmental disorders, Genomic instability, Microsatellite instability, Genome, DNA sequencing, Bioinformatics

## Abstract

Expanded CGG-repeats have been linked to neurodevelopmental and neurodegenerative disorders, including the fragile X syndrome and fragile X-associated tremor/ataxia syndrome (FXTAS). We hypothesized that as of yet uncharacterised CGG-repeat expansions within the genome contribute to human disease. To catalogue the CGG-repeats, 544 human whole genomes were analyzed. In total, 6101 unique CGG-repeats were detected of which more than 93% were highly variable in repeat length. Repeats with a median size of 12 repeat units or more were always polymorphic but shorter repeats were often polymorphic, suggesting a potential intergenerational instability of the CGG region even for repeats units with a median length of four or less. 410 of the CGG repeats were associated with known neurodevelopmental disease genes or with strong candidate genes. Based on their frequency and genomic location, CGG repeats may thus be a currently overlooked cause of human disease.

## Introduction

Repetitive DNA tracts make up a significant portion of the human genome. Short tandem repeats (STRs) are defined as DNA motifs typically ranging from 1 to 6, usually repeated between 5 to 200 units in tandem. In total, these repeats account for 3% of the total human genome^1,2^. Presently, over 30 genetic disorders have been identified resulting from STR expansions^3^. CGG repeats form a specific STR subcategory, associated with human disease, through two distinct mutational mechanisms. The principle examples of CGG repeat expansions disorders are the fragile X syndrome and the fragile X associated tremor/ataxia syndrome (FXTAS)^4,5^. The fragile X syndrome is a frequent syndromic form of intellectual disability (ID) and autism, caused by an expansion of the CGG-repeat in the 5-’UTR of the fragile X mental retardation 1 (*FMR1*) gene, into the full mutation range (> 200 repeats). The expansion triggers an epigenetic mechanism of DNA methylation and additional chromatin remodelling culminating in the transcriptional silencing of *FMR1*. Interestingly, when the repeat is present in the “premutation range” of 55 to 200 units, carriers do not develop fragile X syndrome but are at risk for the late-onset neurodegenerative disorder, FXTAS. Patients present with cerebellar gait ataxia intentional tremor and other neurological symptoms. Brain atrophy, white matter disease, and hyperintensities of the middle cerebellar peduncles can also be present. The disease mechanism of FXTAS is unclear but likely to involve sequestering of RNA’s to the elongated CGG repeat and repeat-associated non-AUG (RAN) translation. Interestingly, several lines of evidence suggest that FXTAS is a consequence of the CGG-repeat elongation per se, independent of its location within the *FMR1* gene. Furthermore, in females, the FMR1 premutation range repeat is associated with fragile X-associated primary ovarian insufficiency (FXPOI), categorized by ovarian insufficiency and hormonal irregularities^6^. Many FXPOI patients report higher indices of thyroid-related issues, depression and anxiety^6^.

Historically, CGG-repeat expansions present as characteristic breaks or gaps in karyotypes obtained from blood cells under folate-deprived conditions^7^. Apart from FRAXA, associated with the fragile X syndrome, 21 additional so-called fragile sites of this rare, folate-sensitive type have been described^8–10^. In the 1970s, karyotyping under folate-deprived conditions was a routine diagnostic procedure for patients with ID. Some studies reported an elevated prevalence of fragile sites in patient populations, with a cumulative frequency of more than 1%, which was not observed in control populations^11,12^. In the decades to follow, 10 of these fragile sites have been cloned as an expanded CGG repeat and more than half have been at least tentatively associated with neurodevelopmental disease. For instance, a CGG repeat located close to the repeat expanded in the fragile X syndrome was found to be associated with a non-syndromic form of ID, shortly after the discovery of the fragile X syndrome^13,14^. In addition, FRA2A has been associated with developmental delay/ID^15^, FRA7A with autism, FRA11B with Jacobsen syndrome^16^, FRA12A with ID^17^ and, most recently, FRA16A with Baratela-Scott syndrome^18^ (see Table 1).

**Table 1:** Overview of the confirmed rare folate-sensitive fragile sites and their associated CGG-repeats.

Fragile site	Gene	Region	Cohort polymorphism	Reference units	Median units	Minimum units	Maximum units
FRA2A	*AFF3*	Intronic	95%*	12	16	1	64
			99%	4	15	1	43
		5′-UTR	12%	8	9	4	47
FRA7A	*ZNF713*	* 5′-UTR	81%	12	13	5	53
		Intronic	92%	21	18	1	53
FRA10A	*FRA10AC1*	5′-UTR	72%	8	9	5	115
FRA11A	*C11orf80*	Exonic	48%	8	10	4	62
FRA11B	*CBL*	5′-UTR	88%	11	13	6	52
FRA12A	*DIP2B*	5′-UTR	97%	12	14	8	120
FRA16A	*XYLT1*	Upstream	95%	4	43	1	83
FRAXA	*FMR1*	5′-UTR	100%	20	28	4	52
FRAXE	*AFF2*	5′-UTR	77%*	28	30	1	59
			34%	4	5	5	55
FRAXF	*TMEM185A*	5′-UTR	61%	10	12	3	55

*In the case of multiple repeats, the repeat indicated as causative of the associated folate sensitive fragile site.

We hypothesized that CGG-repeat expansions are more prevalent in the human genome than currently appreciated and that these expansions might have a role in neurodevelopmental disease. Using recently developed genome-wide STR genotyping tools, we catalogued thousands of CGG-repeats that display characteristics similar to known disease-causing CGG-repeats. Moreover, hundreds of these are associated with known ID/autism genes. Our findings thus raise further questions about the biological role of CGG-repeats and what risk they may pose concerning genetic disease.

## Results

### Genome-wide identification of CGG short tandem repeats

**Figure 1:**
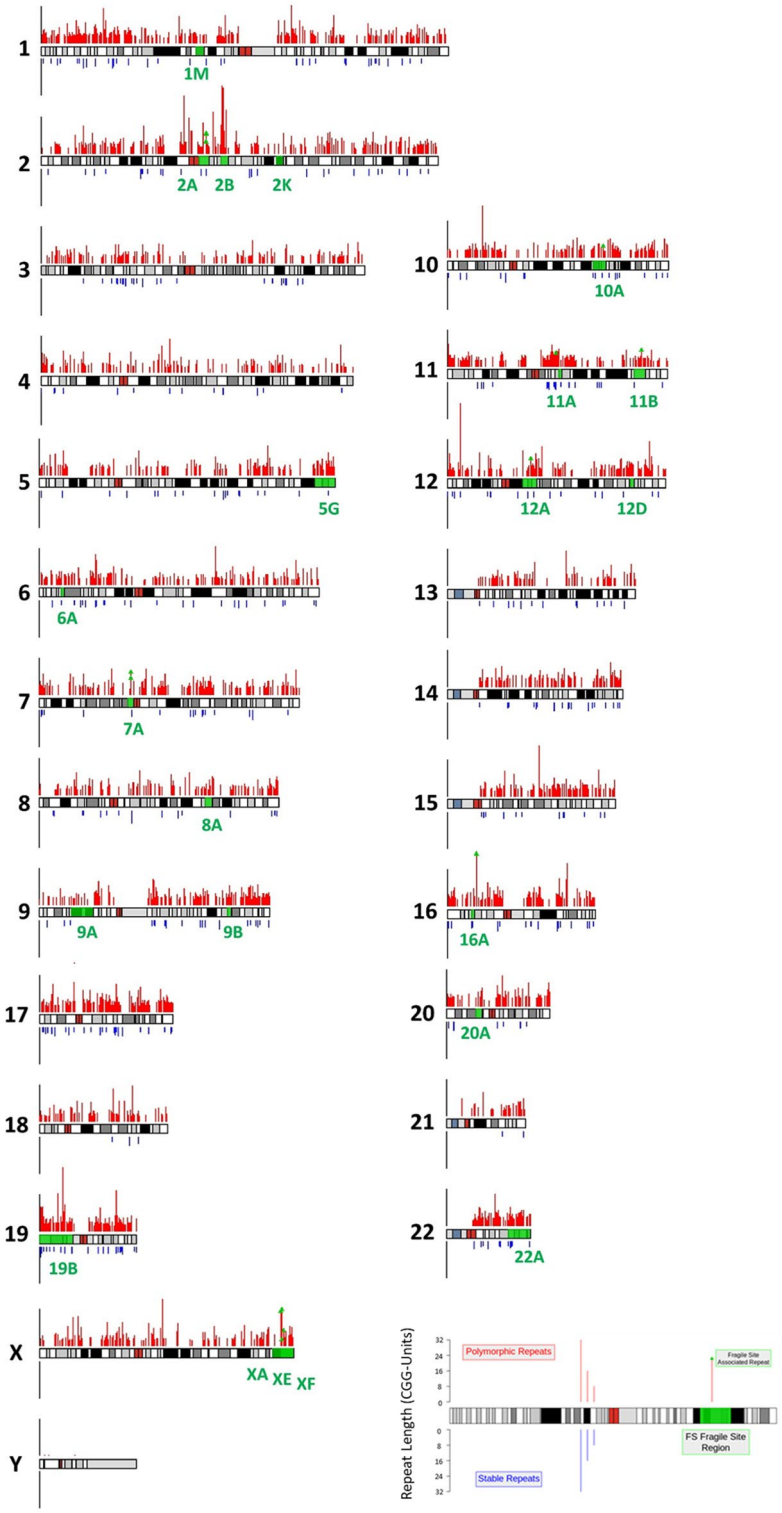
Ideogram of the distribution of all detected CGG short tandem repeats throughout the human genome. Red bars above the chromosome represent the position and size of polymorphic CGG STRs (n = 5683) while the blue bars below represent the stable CGG STRs (n = 418). Median repeat length is indicated by the bar length and length range is indicated on the Y-axis accompanying each chromosome. Green regions on the chromosomes indicate the cytogenic positions where cytogenetically visible folate-sensitive fragile sites have been previously recorded, with the green text indicating the identity of each fragile site. Green markers on repeat bars indicate CGG STRs which have been previously identified as the causative repeats of the associated fragile site^7^. The y-axis bar length equals 32 on all chromosomes.

**Figure 2:**
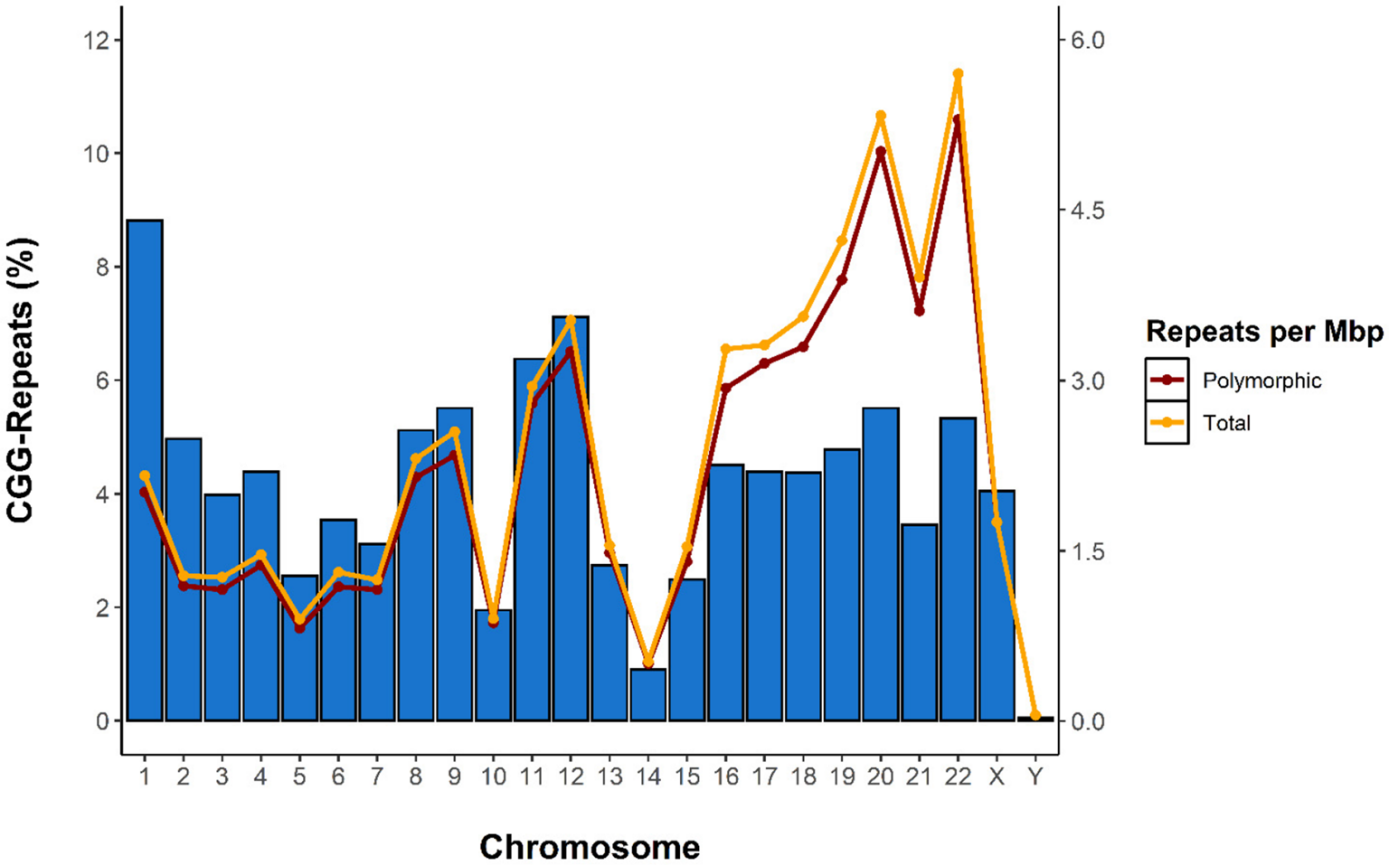
Distribution and density of CGG short tandem repeats per chromosome. Percentage total of CGG STRs per chromosome are represented by the blue bars. Repeat density per chromosome (repeats per Mbp) for polymorphic (n = 5683) and total (n = 6101) CGG STRs are represented by red and orange lines respectively.

Using the Tandem Repeats Finder algorithm to extract repetitive sequences from the GRCh37/hg19 reference genome, we retained a total of 6110 CGG-repeats with a length of 4 or more units. The repeats are unequally distributed across the genome (Figs. 1 and 2), with densities ranging from 0.52 to 5.7 repeats per Mb, for chromosomes 14 and 22 respectively. Interestingly, the Y chromosome is virtually devoid of CGG-repeats.

**Figure 3:**
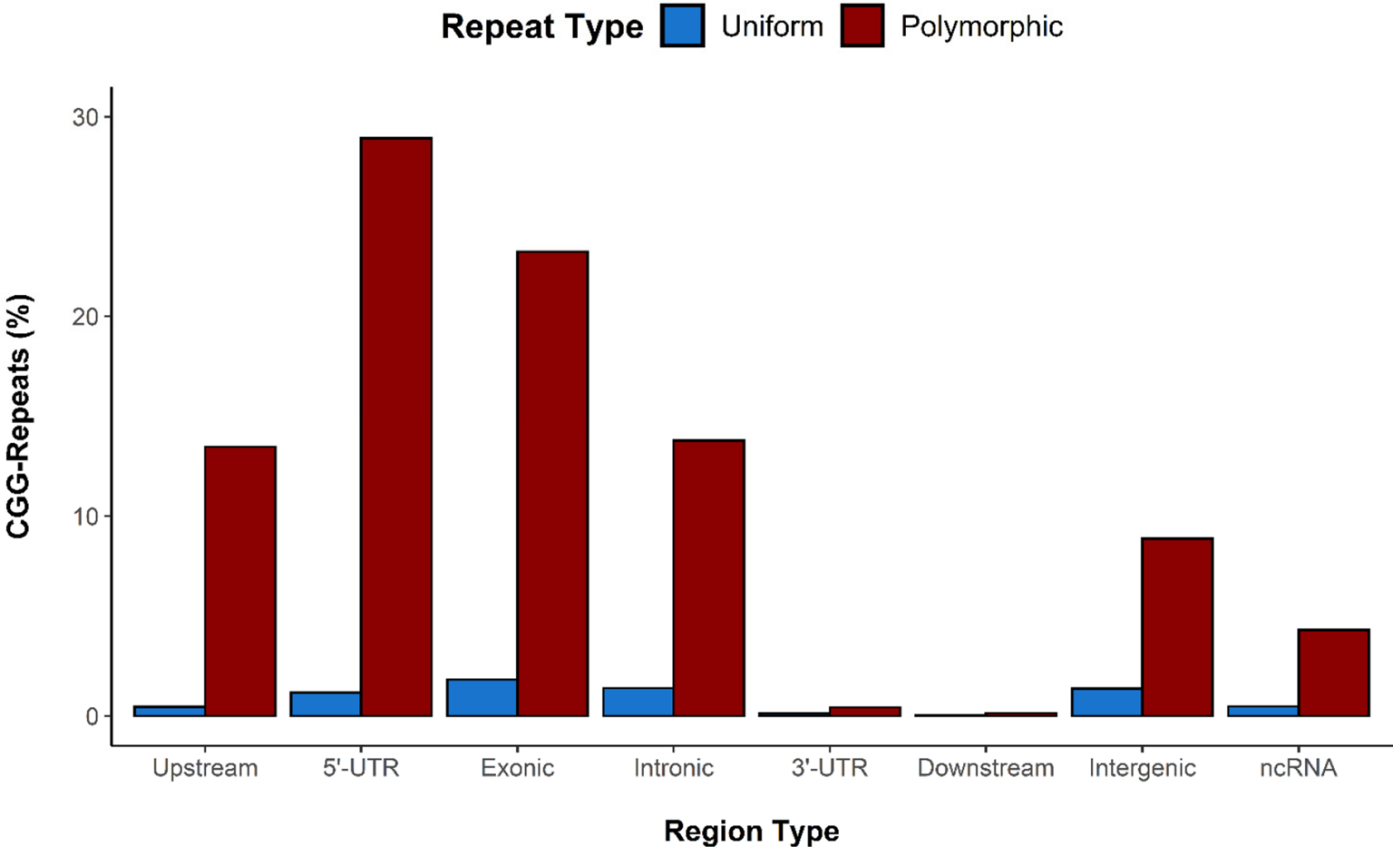
Occurrence of stable and polymorphic CGG short tandem repeats throughout the different genetic regions. Percentage total of uniform and polymorphic CGG STRs by genetic region. Upstream (< 1 kb from start of gene) (n = 849), 5′-UTR (n = 1836), Exonic (n = 1529), Intronic (n = 925), 3′-UTR (n = 34), Downstream (< 1 kb from end of gene) (n = 11), Intergenic (> 1 kb from gene) (n = 625), and ncRNA (n = 292).

In total, of the 6110 repeat loci in our experimental data, 8 had to be disregarded due to poor coverage while one repeat locus was disregarded due to the presence of only partial flanking reads. The remaining 6101 repeats were within or in proximity to 4370 different known genes. Of those, 3335 genes contained at least one intragenic CGG-repeat, although many were associated with multiple CGG repeats within or immediately (within 1000 bp) upstream or downstream of the gene. 824 genes contained at least one CGG-repeat within the adjacent intergenic regions. Of these, 211 genes contained both an intragenic CGG-repeat and a CGG-repeat in the surrounding intergenic regions. The majority of CGG-repeats fell within the 5′-UTR gene regions (*n* = 1836/6101), exons (*n* = 1529/6101), or immediately upstream of genes *(n* = 849/6101) (Fig. 3). Interestingly, there was an almost complete lack of CGG-repeats within the 3′-UTR (*n* = 34) and immediate downstream (*n* = 11) regions.

**Figure 4:**
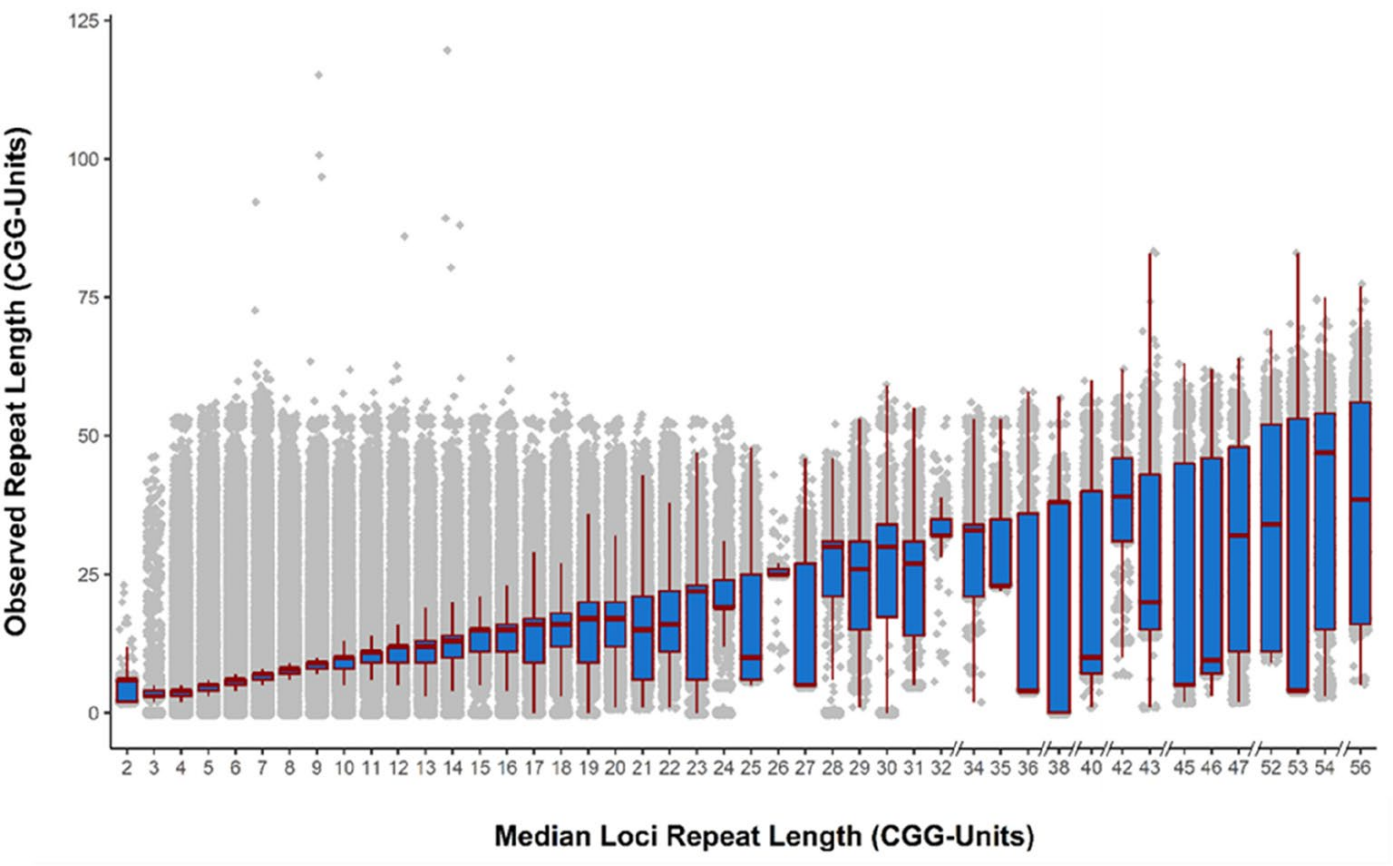
Deviation in length of polymorphic repeats from the median locus repeat length. Here, all observed repeat lengths of all polymorphic repeat alleles were plotted against the respective locus median repeat length. The X-axis is not continuous as at the higher end of median repeat lengths not all sizes were represented. It can be noted that for repeat loci with lower median repeat length, most observed repeat lengths clustered around the median repeat length. However, many of these observed repeat lengths at this range still differed considerably from the median repeat length. In general, observed repeat length variance appeared to increase as the median repeat length increased (Supplementary Table 2).

**Figure 5:**
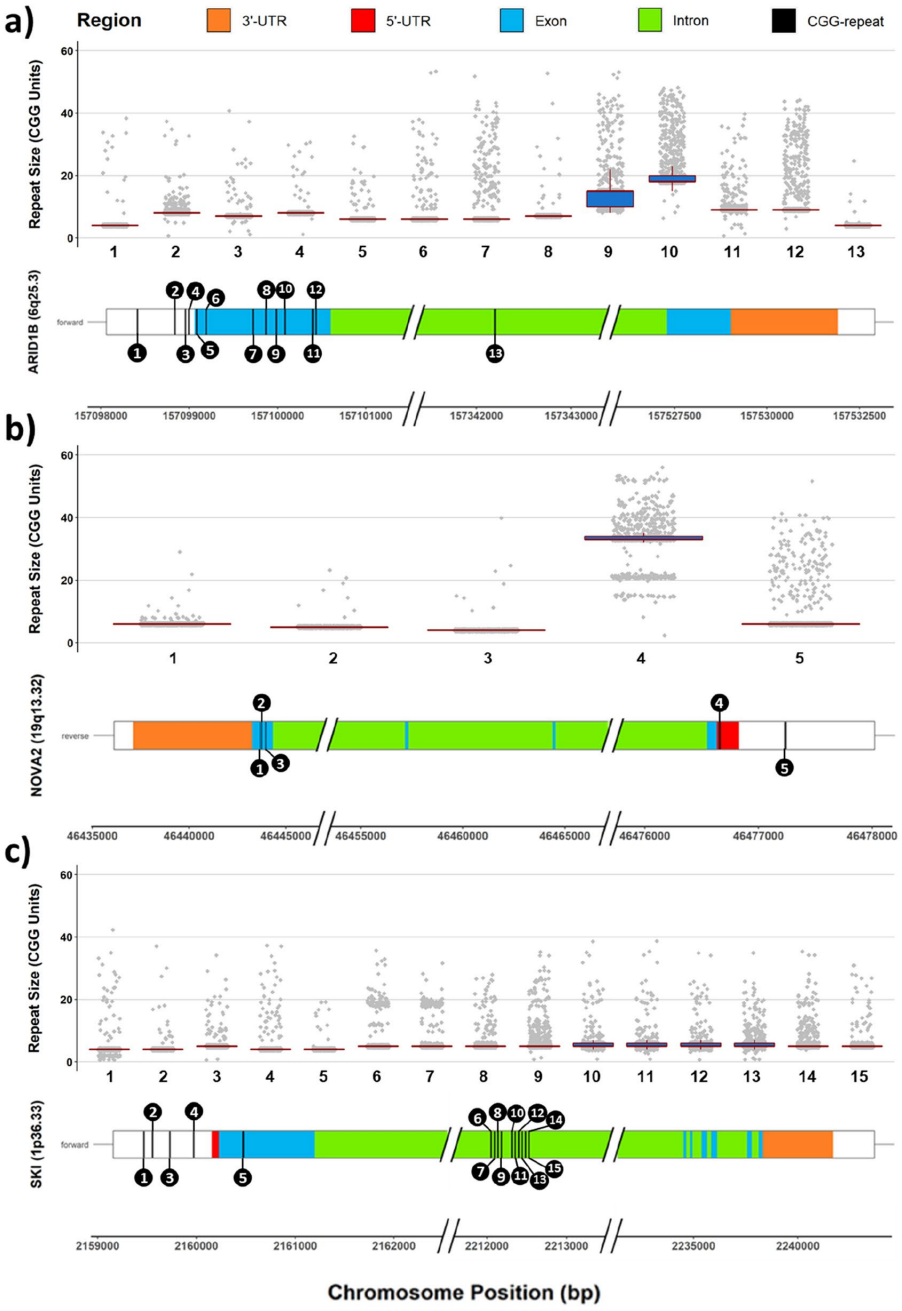
Repeat distribution and repeat length variation in several genes across the control population. All CGG STRs observed in these three genes were polymorphic. (**a**) ARID1B contained 13 CGG STRs. 3 upstream, 1 5′-UTR, 8 exonic, and 1 intronic; (**b**) NOVA2 contained 5 CGG STRs. 1 upstream, 1 5′-UTR, and 3 exonic; and (**c**) SKI contained 15 CGG STRs. 4 upstream, 1 exonic and 10 intronic.

### Genotyping accuracy

Several STR genotypers exist to evaluate repeat lengths in short-read whole-genome sequencing data^19^. To validate the output of the genotypers, we compared the performance of GangSTR^20^ and ExpansionHunter^21^ on a cohort of 100 whole genomes (WGS) samples in detail. There was a 91.22% consistency between the two program suites: 34.92% (*n* = 211,262/605,074) of the called repeat alleles were genotyped identically by ExpansionHunter and GangSTR, while 56.30% (*n* = 340,666/605,074) of the calls differed by a single repeat unit, which was attributed to different handling of incomplete trailing repeat units. The primary reason for the 8.78% genotype discrepancies between both algorithms was related to the inability of GangSTR to account for repeat interruptions, leading to larger repeat size estimations by ExpansionHunter in 6.82% (*n* = 41,274/605,074) of calls. In 0.61% (*n* = 3678/605,074) of calls, GangSTR predicted larger repeat lengths, by compounding reads together and apparently overestimating the length of the repeat. Finally, in 1.35% (*n* = 8194/605,074) of calls, GangSTR failed to genotype repeats while ExpansionHunter did provide a genotype. Experimental validation of some of the repeats in *ABCD3, ACVR2A, LHX5-AS1, SHANK2, RASGEF1C,* and *TMEM268* in a subset of the individuals in our cohort showed that repeat lengths predicted by ExpansionHunter were identical with the repeat lengths measured through PCR amplification and Sanger sequencing (Supplementary Fig. 1). Based on these observations, combined with the ability of ExpansionHunter to estimate repeat lengths beyond the short-read lengths and handle repeat interruptions, we selected this algorithm for our further analyses. Furthermore, as most other STR genotypers also require a repeat coordinate file (based on STR locations in the reference genome) at the initialization of the algorithm, it seems unlikely to us that use of similar STR genotypers over ExpansionHunter would yield a greater detection of CGG-repeats.

### Characterization of allelic polymorphism of the CGG-repeats

PCR-free WGS data was obtained from a control cohort of 544 unrelated adult individuals from the Next Generation Children project (NGC)^22^. Out of the 6110 CGG-repeat sites investigated, nine loci were excluded as none of the WGS samples included spanning or in-repeats reads for those loci. Overall, approximately 99.8% of alleles were genotyped at the remaining 6101 sites. Strikingly, across the cohort, 93.1% (*n* = 5683/6101) of the CGG-repeat loci displayed a least some degree of variability, with only 6.9% (*n* = 418/6101) of the CGG-repeat loci showing uniform size. We noticed that in many cases the observed median repeat lengths were discordant with the repeat length provided by the GRCh37/hg19 reference genome. Therefore, we substituted the repeat length annotated in the reference genome with the total population median repeat length observed in our study cohort. This value, referred to as the median repeat length for the remainder of the study, ranged from 2 to 56 units in our cohort. Although we did not target repeats with a reference length below four units, several loci showed an experimental median population repeat length below our original detection threshold. CGG-repeat loci variation can be observed in Fig. 4, and the full repeat catalogue with observed population characteristics is available in supplementary Table 1. In general, we observed highly variable CGG-repeat characteristics amongst the individuals of our cohort. On average 18.2% (range 13%-34%) of all CGG-repeats in each individual were polymorphic, with the largest repeat observed ranging from 54 to 120 units.

Next, Fig. 4 summarizes to what degree (prevalence) and extent (size) the 5683 polymorphic CGG-repeat loci tend to deviate from the median population repeat length. As expected, repeats exhibiting a lower median repeat length tended to cluster near their expected length, while larger repeats showed a broader variability range. Nevertheless, even loci with a median repeat length of 1–3 units, exhibited significant repeat size polymorphisms. Similarly, the largest allele observed at any locus seemed almost independent of its median repeat length. For instance, the largest allele observed in our cohort consisted of 120 repeat units and was detected at a locus with a median repeat length of 14 units. In contrast, the smallest observed allele for many loci was just a single repeat unit in size. This holds mainly at smaller loci but was also detected in repeats with a median repeat size of up to 43 repeat units. Overall, our results indicate a positive correlation between the median repeat length and the degree of polymorphism across samples (Supplementary Fig. 2). At a median of 4 CGG-units, approximately 85% of all loci displayed a length polymorphism in at least a single individual, while the remaining 15% were non-polymorphic throughout the full cohort. Conversely, approximately 96% of repeats with a median repeat length of 8 units were polymorphic and all repeats with a median repeat length over 12 units were polymorphic.

### Characteristics of the known fragile site associated CGG short tandem repeats

All ten repeats previously cloned based on their association with chromosome fragile sites were detected within our analysis (Table 1). Out of the known disease-associated repeats, the FRA16A/*XYLT1* gene displayed the largest median repeat length in our cohort, with 43 repeat units, while the other fragile site-associated repeats displayed a median repeat length in the range of 5 to 30 CGG-units (Table 1). In terms of the largest fragile site-associated repeat detected among the cohort, one individual displayed a *FRA12A*/*DIP2B* CGG-repeat length of 120 repeat units. Another individual displayed a repeat of 115 repeat units in the 5′-UTR of the *FRA10AC1* gene (Table 1).

Interestingly, in several of the genes associated with a fragile site, we observed multiple CGG-repeats. FRA2A/*AFF3* was observed as containing three separate CGG-repeats, while FRA7A/*ZNF713* and FRAXE/*AFF2* both contained two repeats. Although one of the repeats in the *AFF3* gene displayed a relatively low polymorphism rate and a short median repeat length, all three repeats displayed the ability to expand by a considerable length. This was also observed in the *AFF2* gene, where the second repeat, with a polymorphism rate of 34% and a median repeat length of 5, was seen to expand as high as 55 repeat units. Illustrating the functional variability of repeats, it is the intronic repeat in AFF3 (Median Units: 16) which, has been credited as the causal repeat behind FRA2A, while in *ZNF713* it is the 5′-UTR repeat which is reported as the cause of FRA7A^15,23^. In addition, the repeat with the largest median repeat length was 56 CGG-units long and located in the intergenic space between the genes *RGPD5* and *RGPD6* in the 2q13 cytoband, where the 2B folate-sensitive fragile site is located.

In analogy with the paralogous genes *AFF2* and *AFF3* that are both preceded by a CGG repeat, other gene families appeared to be highly associated with CGG-STRs. An example is the family of forkhead box (FOX) genes. The *FOXP1* gene is a gene commonly linked to ID, and we detected two CGG-STRs within this gene. No less than 75 CGG repeats in total were observed associated with its paralogues. Several paralogues contained multiple repeats, such as *FOXD4L5* (9), *FOXC1* (6), *FOXD1* (6), *FOXD4L4* (4), and *FOXG1* (3), including several large and highly unstable repeats.

### Functional annotation of CGG-repeat-associated genes

To investigate a possible correlation with neurodevelopmental disease, we investigated the characteristics of genes associated with CGG repeats apart from those associated with known fragile sites. We compared the 4370 CGG-linked genes with a list of 1295 ID genes used in our centre for routine screening for ID and related neurodevelopmental disease. We highlighted 410 genes associated with neurodevelopmental disease. We observed a significant enrichment of known ID genes within the CGG-associated genes (chi-square; p < 2.2e-16; Table 2), with at least 26% of all established ID genes containing 1 or more CGG repeats. ID genes were classified as either repeat-associated or non-repeat-associated. Therefore, the number of repeats associated with each gene did not affect our analyses.

**Table 2:** Results of Chi-squared tests for 410 ID-associated genes that contained one or more CGG STR which were present in a list of 1295 genes used for clinical screening of ID.

Repeat location	Number of Genes	X^2^	*p* value
5′-UTR	241	240.44	1.244e−6
3′-UTR	7	23.507	< 2.2e−16
Upstream	138	107.89	< 2.2e−16
Intronic	164	178.28	< 2.2e−16
Exonic	184	152.42	< 2.2e−16
Total*	410	264.05	< 2.2e−16

*The total number of genes is equal to 410, however as some genes contained multiple repeats within different regions some genes are represented in multiple region subsets.

Interestingly, 57% of genes containing a polymorphic CGG-repeat (*n* = 2017/3546), displayed a low haploinsufficiency index of less than 40% and 46% (*n* = 1621/3546) of these genes displayed a pLi score greater than 0.75. Both these scores indicate a likely intolerance to a reduction in expression following silencing of one of the present alleles, making these genes potential candidates for further investigations into CGG-expansion induced silencing and functional disruption.

A more general functional evaluation of CGG-linked genes was conducted using the PANTHER Classification System. A total of 3864/4370 CGG-associated genes were mapped to the PANTHER database. Based on these genes, enrichment analysis was performed on 4255 Gene Ontology hits for biological processes, 3110 for molecular function, 2686 for cellular components, 2446 for protein class, and 1944 for pathway involvement. In all categories, we observed a diverse set of associated roles (Supplementary Fig. 3). Of interest was the relatively high occurrence of items related to transcription regulation, including transcription factors (protein class; 16.4%), and transcription regulation (molecular function; 12.8%). Amongst the 114 different biological roles identified, most pathways represented less than 1% of the genes. In contrast, the Gonadotropin-releasing hormone receptor pathway and the Wnt signalling pathway were both associated with 5.5% of the genes. The CCKR signalling map, Angiogenesis, and Integrin signalling pathway made up 3.8%, 3.1%, and 3% of the results respectively.

## Discussion

We applied Tandem Repeats Finder to build a catalogue of CGG-STRs in the human genome. In total, we confirmed 6101 unique loci, including all 10 CGG repeats associated with known fragile sites. We characterized this specific class of STR using ExpansionHunter in a control cohort of 544 unrelated individuals. The overall allele size heterogeneity of the CGG repeats is astonishing. We found that 93.1% of the loci show length polymorphisms. Median repeat lengths ranged from 2–56 repeat units, with individual genotypes ranging from 1 to 120 repeat units. Even repeat loci with a median repeat length as low as 4 repeat units showed alleles of over 50 repeat units in some individuals, although overall in our cohort analysis the majority of larger alleles were related to loci with higher median repeat sizes. Repeats induce unusual DNA structures that can result in strand slippage during both meiotic and mitotic DNA replication, repair and recombination^24^. It is therefore likely that the observed allelic heterogeneity is a result of replication errors in germline cells or their precursors in previous generations.

A feature discriminating CGG-repeats from other STRs is their predominant location with respect to genes. While STR in general are relatively equally distributed over the genome over 50% of CGG-repeats were detected within exonic and 5′-UTR regions, in line with earlier observations that demonstrated that CG-rich repeats are overrepresented in the exonic regions^25,26^. This is in line with previous studies reporting predominance of CGG-repeats in promoter regions in humans, and even in yeast^27,28^. While it has been reported that the Y chromosome displays STR densities comparable with the other chromosomes, both this study and Subramanian and colleagues indicate this is not the case for CGG STRs as the Y chromosome was virtually depleted of CGG-repeats in our study^25^. It has been reported that the length of individual STR-alleles may play a role in the expression of various complex traits^29,30^ and as CGG-repeats are preferentially located at or near promotor sites, it can be speculated that the variability in length we detected potentially plays a role in gene-expression regulation. Another hint of the functional relevance is the conservation of CGG repeats across gene families, such as the *AFF* and *FOX* families of genes. At this stage, it is not known whether these CGG-repeats were present in the single common ancestral gene and were retained in some paralogues but lost in others or whether the repeat arose independently after the evolution of the paralogous genes. Several studies have explored cross-species differences and impacts of STRs. While many repeat loci are conserved across mammalians their characteristics differ depending on the species^31–33^. Orthologous repeat loci often differ in polymorphisms, repeat length, and repeat interruptions based on the species in question^30,31,33^. Future studies specifically into the conservation of CGG STRs across species may shed light onto the biological role of these rare repeats.

Further highlighting the potential clinical relevance of CGG expansions, we saw a significant overrepresentation of CGG-repeats in neurodevelopmental disease genes, with 410 of the 1295 genes of an in-house neurodevelopmental disease diagnostic panel being associated with a CGG repeat (*p* < 2.2e-16; Table 2). One of these genes, *ARID1B,* causes Coffin-Siris syndrome, an autosomal dominant form syndromic ID and autism^34^. Strikingly, we observed 13 unique CGG-repeats associated with *ARID1B*, three immediately upstream of the gene, one in the 5′-UTR, eight exonic and one intronic (Fig. 5). Presence of multiple repeats within a single gene is a common feature, seen in 1097 genes. For *ARID1B,* all of the CGG-repeats were polymorphic in our cohort. Interestingly, repeat characteristics for *ARID1B* were in line with observations from repeats in known fragile sites, which show a median repeat length of 5–43, can range from 1 to 120 repeats and have polymorphism rate of 48% to 100% (Table 1). For example, the size of the largest exonic repeat ranged from 8–53 CGG repeats with a median size of 20 units and a polymorphism rate of 41%, and the adjacent repeat exhibited a maximum repeat length of 48 units and polymorphism rate of 97%.

Combining the association to known neurodevelopmental disease genes and the observed overall length variability of CGG-repeats with their predominant location in regulatory regions, the abundance of repeat-associated genes involved in transcription regulation, revealed by the GO-term analysis and the predicted haploinsufficiency of the associated genes, we hypothesize that many CGG-associated genes may be candidate genes for disease, e.g. *NOVA* alternative splicing regulator 2 (*NOVA2*) gene. We observed that within our cohort *NOVA2* was associated with five unique polymorphic repeats, 4 of which are polymorphic in our control dataset (Fig. 5). The largest repeat fell within the 5′-UTR, displayed a median repeat length of 36 units and an 87% polymorphism rate. It also displayed one of the larger repeat lengths observed within our cohort, with a length of 56 units. In line with our suspicion, the Developmental Disorders Genotype–Phenotype Database (DDG2P) has flagged *NOVA2* as being a probable cause of ID as a consequence of a loss of function event. Future analysis needs to determine whether repeat expansions in these genes occur and contribute to disease.

We observed a “soft-limit” to the length of observed CGG STRs at approximately 50 CGG units (Fig. 4). This may be due to both the biological nature of CGG STRs and/or a technical limitation of ExpansionHunter. As pathogenic effects may manifest in an individual when a CGG extends into the pre-mutation range, this may be why CGG repeats beyond 50 units are rare. However, this phenomenon would require confirmation through further investigations. The likely reason for the observation of the CGG STR soft-limit is that ExpansionHunter relies on the presence of fully in-read repeats to estimate when STR size has extended beyond read length. Therefore, if a trinucleotide repeat has extended beyond read length (150 bp), but no full in-read repeats are present, then the algorithm could estimate the repeat length at a maximum of 50 units. It would only be when a repeat has extended considerably past read-length that multiple in-read repeats would be consistently detected and that ExpansionHunter could report a repeat length beyond 50.

Interestingly, a recent report observed premutation-ranged CGG-repeat expansions in the *LOC642361/NUTM2B-AS1*, *LRP12*, and *NBPF9* genes. The associated disorders share a striking clinical similarity to FXTAS^35–39^. The similarities between the clinical manifestations of these disorders add further evidence to the theory that the pathological characteristics of expanded CGG-repeats are being replicated elsewhere in the genome, independent of the associated gene. The replication of STRs elsewhere in the genome resulting in similar pathogenic characteristics has been observed in other repeat expansion disorders. For example, familial adult myoclonic epilepsy which is caused by an expanded TTTTA/TTTCA repeat has been detected at multiple locations throughout the genome^40^. Our study predicts that premutation sized CGG-repeats may be considerably more common than currently considered. Given the relatively low yield of screening studies for *FMR1*-associated CGG-repeats in the premutation range in clinically relevant patient populations, it could be recommended to rescreen these same populations for all repeats in our CGG-catalogue for potential disease-causing expansions.

While our work was under review, a study conducted by Trost et al.^41^ was published that investigated STRs of 2–20 bp in a genome-wide setting and in relation to autism spectrum disorder^41^. In our study, we detected a much higher number of CGG STR loci than Trost and colleagues, as the emphasis of their work was on expanded STR loci utilizing ExpansionHunter Denovo^42^. This algorithm only detects repeat expansions that exceed the length of the sequencing reads, typically 150 bp, corresponding to 50 CGG repeat expansions. Similarly, to Trost and colleagues, we also detected CGG repeats displaying considerable size and polymorphisms at all cytogenetically visible folate-sensitive fragile sites. These repeats are prime candidates for the causative element of these fragile sites and are previously unreported before these studies.

In conclusion, we characterized 6101 CGG-repeats in the human genome, of which over 90% are polymorphic in our reference set of 454 individuals, a percentage that is likely to rise should additional populations be screened. A potential limitation of this study is the reliance on a reference genome to construct the baseline STR panel^43^. We demonstrated that many repeats could contract to less than 4 repeat units of length. Therefore, any repeat loci contracted, deleted, or misaligned in the samples used to construct the reference genome would be missed in the analyses similar to our study. Although one would expect that these repeats form a minority in our analysis, it would be interesting to evaluate future and more accurate high quality de novo genome assemblies for the presence of additional CGG repeats.

About two-thirds of the CGG-repeats are at or in the vicinity of gene promoters, including hundreds of genes known to be associated with a neurodevelopmental disease. Additional genes include many candidate genes for neurodevelopmental disease on the basis of gene-ontology analysis and haploinsufficiency prediction scores. Our study in a control population has not revealed expansions into a potentially pathogenic range, but repeats within the premutation range were observed, and hundreds of CGG-repeats demonstrated characteristics similar to those observed in repeats associated to fragile sites, that have been proven to expand. Additionally, we gained insight into the unexpected behaviour of CGG-repeats with lower median repeat sizes, where we noticed expansions nearing the premutation range. Furthermore, as the pathological mechanism of the CGG-repeat in neurodegenerative disorders such as FXTAS is not well understood, our study raises questions about the biological role of CGG-repeats in general, and about the risk, they may pose with regard to evolution and genetic disease.

## Methods

### Genome-wide tandem repeat panel construction

The genomic location of all short tandem repeats of 4 units and higher present in the GRCh37/hg19 human reference genome assembly was derived by applying the Tandem Repeats Finder algorithm (version 4.09) to a FASTA file of the hg19 genome^44^. The parameters used were MatchScore: 2, MismatchScore: 5, IndelScore: 17, MaxPeriod: 20, PM: 80, PI: 10, MinScore: 24, and MaxLength: 1000. The resulting panel was then filtered for all reading frame variants of CGG-repeats (GCC, CCG, GGC, CGC, GCG). The resulting repeat panel was then filtered further utilizing pairwise2 from the BioPython (version 1.75) library to conduct local pairwise alignments to determine the longest repeats with least interruptions within the reference genome regions identified through Tandem Repeats Finder. The parameters used for pairwise2 were x: 1/2, m: − 2, d: − 5, and c: − 1. The resulting repeat panel consisted of 6110 distinct CGG-repeat loci.

### Whole genome sequencing

PCR whole-genome sequencing (WGS) of 100 individuals with no known repeat disorders or repeat expansions were included in this study. WGS was conducted by BGI (Shenzhen, Guangdong, China) as previously described^45^. All clean reads were aligned to the GRCh37/hg19 reference genome using the Burrows-Wheeler Aligner (BWA v0.7.12). Picard-tools (v1.118) was used to sort the alignments by coordinate and subsequently convert and store the genome alignment as BAM files.

PCR-free WGS was conducted on DNA samples obtained from blood of 544 non-affected individuals from the NGC cohort^22^. DNA samples were shipped to Illumina (UK) for sequencing and were prepared with the Illumina TruSeq DNA PCR-Free Sample preparation kit (Illumina, Inc) as previously described^22,46^. Samples were sequenced on the Illumina Hiseq 2500, and quality control and read alignment to the human reference genome GRCh37/hg19 was performed by Illumina as previously described^22,47^. Average coverage obtained was 30–40 × for the nuclear genome and 800–1000 × for the mitochondrial genome.

### Genome-wide STR analysis utilizing GangSTR and ExpansionHunter

Two separate short tandem repeat genotyping algorithms were utilized, namely GangSTR (version 2.0) and ExpansionHunter (version 3.1.2), developed by Mousavi et al.^20^ and Dolzhenko et al.^21^ respectively. For both ExpansionHunter and GangSTR, the default parameters were used, and the same hg19 FASTA file was used for the genome reference arguments. For the GangSTR “–regions” and the ExpansionHunter “–variant-catalog” arguments, custom BED and JSON files were generated respectively, based on the CGG repeat panel constructed by Tandem Repeats Finder, as described in the above section. The VCF output files of both algorithms were then parsed to extract the STR data using the PyVCF (version 0.6.8) library in a Python 3.6.8 environment. ExpansionHunter and GangSTR were applied to the 100 control WGS samples, sequenced by BGI, for comparison of genotyper accuracy. ExpansionHunter was applied to 544 control WGS samples, sequenced by Illumina, to obtain the repeat characteristics reported in this study. An R (version 3.6.3) environment was used for downstream data analysis of the STR data. Data was analysed and summarised by repeat locus, chromosome, and sample. A repeat at a given locus was defined as polymorphic if at least one individual displayed a repeat length which differed from the median repeat length, at that given locus, of the screened population. The polymorphism rate of a repeat locus was defined as the proportion of alleles which displayed a polymorphic repeat length throughout the screened cohort.

### Gene annotation

Gene-based annotation of the detected CGG short tandem repeat (CGG-STR) loci was carried out using the software tool ANNOVAR and the refGene hg19 gene database^48^. If a repeat fell outside of the gene body, it was annotated as being “upstream” or “downstream” if they were located within 1 kb of the start of the 5′-UTR or the end of the 3′-UTR of the gene in question. If a repeat was located over 1 kb from a gene, it was defined as “intergenic”. Haploinsufficiency and pLI score data per gene was obtained from the DECIPHER and ExAC databases respectively^46,49^. The PANTHER Classification System (v14.1) (Gene Ontology Phylogenetic Annotation Project) was used to facilitate a high-throughput gene ontology analysis of the genes identified by our STR analysis and gather functional and molecular characterization data of the genes associated with CGG-repeats.

### Repeat length validation by Sanger sequencing

Six genes within the folate-sensitive fragile site regions were selected for validation of the predicted ExpansionHunter repeat lengths. PCR amplification and Sanger sequencing were used to validate called repeats lengths in 8 individuals from our control cohort. PCR reactions were prepared using GoTaq polymerase and reagents (Promega, Leiden, Netherlands). PCR reactions were run on a Veriti 96 Well Thermal Cycler (Applied Biosystems, Foster City, CA, USA). PCR products were sequenced by use of an ABI Prism 3130 Genetic Analyzer (Applied Biosystems, Foster City, CA, USA). The DNA primers were supplied by Integrated DNA Technologies, Leuven, Belgium.

## Supplementary Information

Supplementary Information.

Supplementary Table 1.

Supplementary Table 2.

Supplementary Table 3.

Supplementary Table 4.

## Data Availability

Most data generated or analysed during this study are included in this published article or in the supplementary information files. The other datasets used and/or analysed during the current study are available from the corresponding author on reasonable request.
