# Supplementary material for: Abundancy of polymorphic CGG repeats in the human genome suggest a broad involvement in neurological disease
Source: Sci Rep. 2021 Jan 28;11:2515. doi: 10.1038/s41598-021-82050-5 (PMC7844047; doi:10.1038/s41598-021-82050-5)
Supplement: Supplementary file 1 — Supplementary Information. [file 41598_2021_82050_MOESM1_ESM.pdf]

Abundancy of polymorphic CGG repeats in the human genome suggest a broad involvement in neurological disease

Dale J. Annear[1], Geert Vandeweyer[1], Ellen Elinck[1], Alba Sanchis-Juan[2,3], Courtney E French[4], Lucy Raymond[2,5], *R. Frank Kooy[1]

[1]Department of Medical Genetics, University of Antwerp, Antwerp, Belgium

[2]NIHR BioResource, Cambridge University Hospitals NHS Foundation Trust, Cambridge Biomedical Campus, Cambridge, CB2 0QQ, UK

[3]Department of Haematology, University of Cambridge, NHS Blood and Transplant Centre, Cambridge, CB2 0PT, UK

[4]Department of Paediatrics, University of Cambridge, Cambridge, CB2 0QQ, UK

[5]Department of Medical Genetics, Cambridge Institute for Medical Research, University of Cambridge, Cambridge, CB2 0XY, UK

*Corresponding author:

Department of Medical Genetics, University of Antwerp, Antwerp, Belgium

Tel.: +332759760

E-mail: frank.kooy@uantwerpen.be

Content:

Supplementary Figures

- Supplementary Figure 1. Validation of select CGG-Repeats by PCR amplification and Sanger sequencing.
- Supplementary Figure 2. Relationship between median CGG-repeat length and repeat polymorphisms.
- Supplementary Figure 3. Percentage of involvement of CGG repeat associated genes with GO term defined A) molecular functions and B) protein classes.

Supplementary Tables

- Supplementary Table 1. Summary of all CGG-repeat loci detected by ExpansionHunter and their corresponding attributes (*n*=6901).
- Supplementary Table 2. Median repeat lengths observed among all polymorphic CGG repeat loci (n=5673) and the corresponding number of repeat loci that displayed each median repeat length.
- Supplementary Table 3. Genes of interest. Genes associated with ID, AD, and CGG-repeats with their corresponding haploinsufficiency index and pLI score **(***n*=410**).**
- Supplementary Table 4. List of genes used genes used for routine screening for ID and related NDD (*n*=1295)

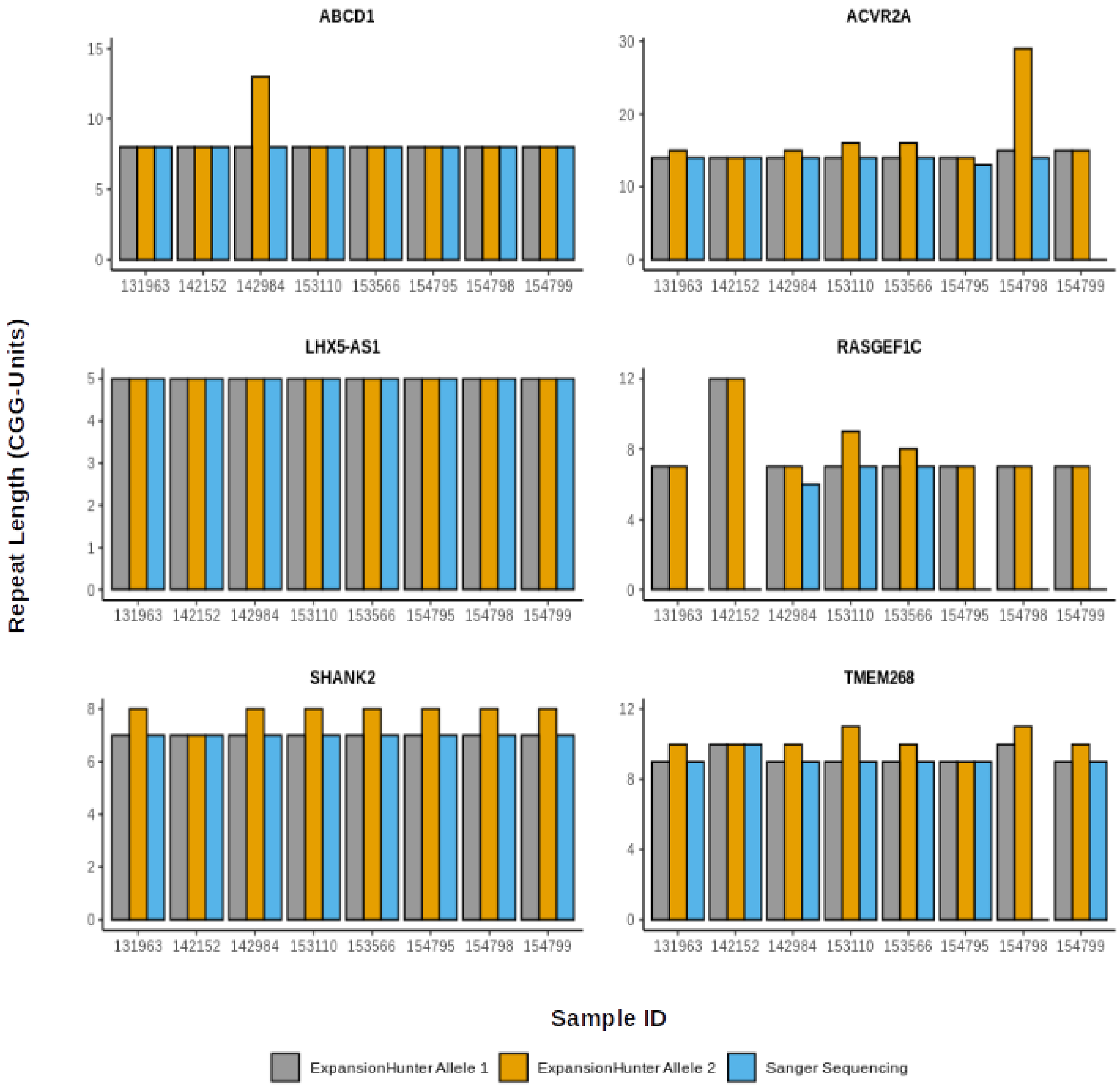

Supplementary Figure 1. Validation of select CGG-Repeats by PCR amplification and Sanger sequencing. Comparison of ExpansionHunter repeat length predictions with observed length of Sanger sequenced repeats. For homozygous alleles ExpansionHunter predictions were identical to sequenced repeats. For heterozygous alleles, only the size of smaller allele could be elucidated. However, these we all identical with ExpansionHunter reports.

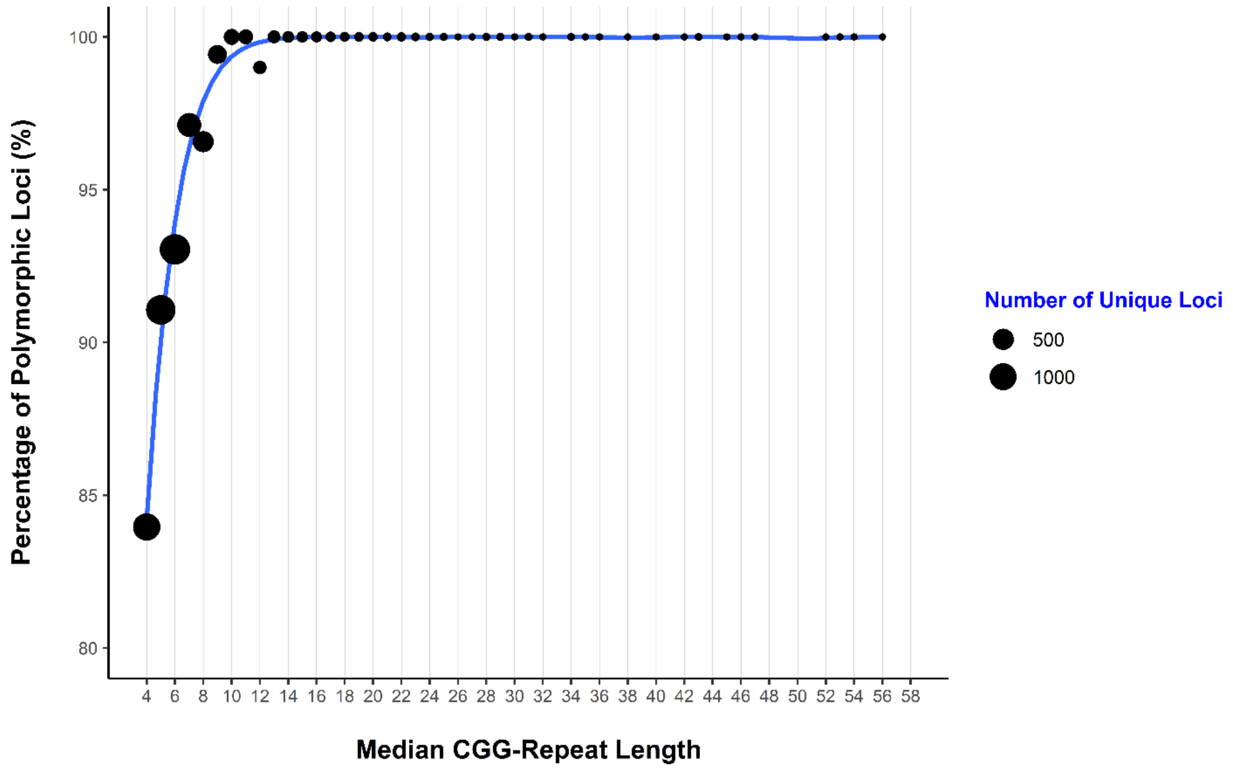

Supplementary Figure 2. Relationship between median CGG-repeat length and repeat polymorphisms. As median repeat length increases so does the proportion of repeat loci with that median repeat length that are polymorphic. All STR loci with a median repeat length equal or greater than 12 CGG units were polymorphic among the tested cohort.

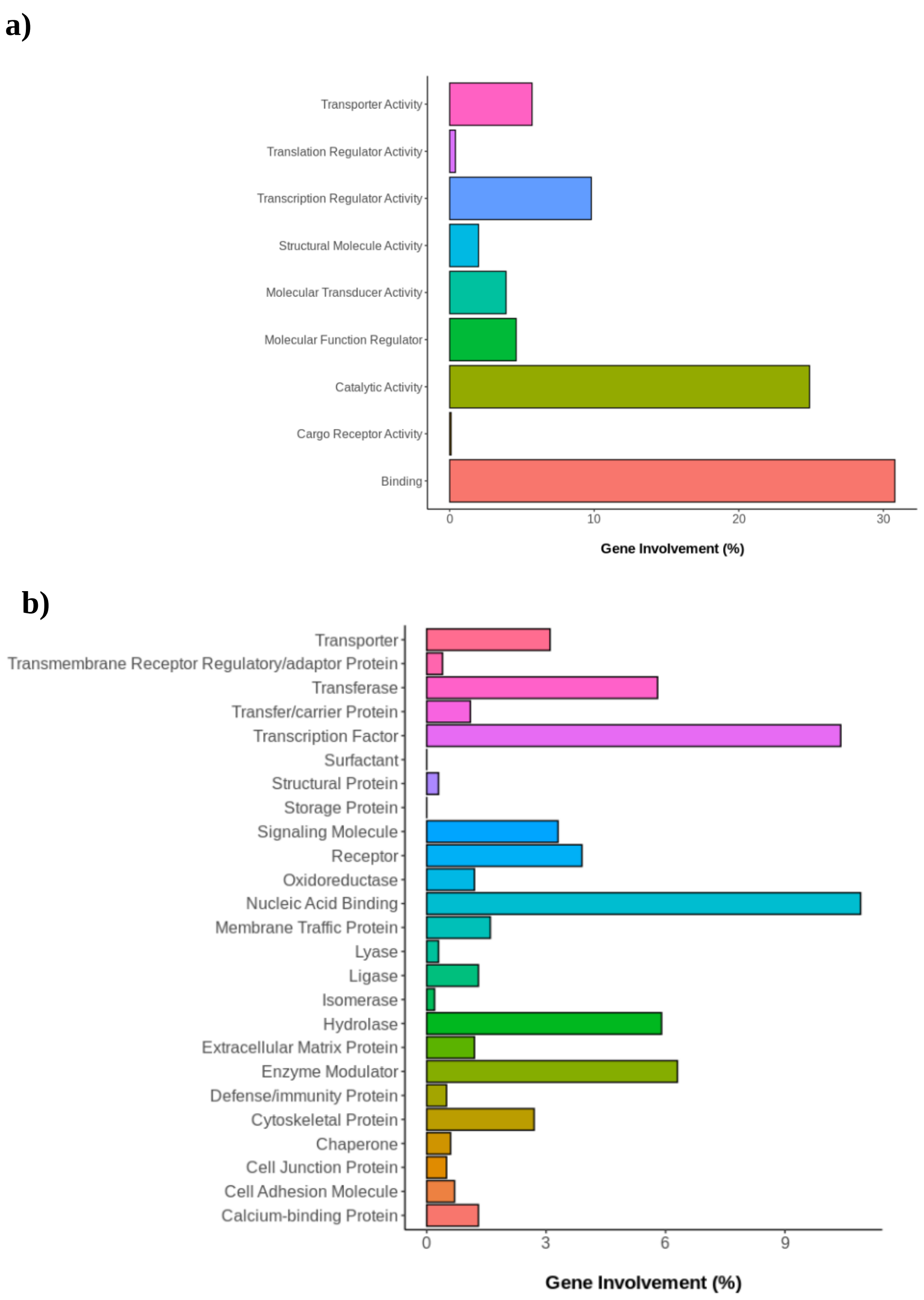

Supplementary Figure 3. Percentage of involvement of CGG repeat associated genes with GO defined terms. a) molecular functions and b) protein classes.

Supplementary Table 1. Summary of all CGG-repeat loci detected by ExpansionHunter and their corresponding attributes (*n*=6101).

See the attached txt file Supplementary Table 1.txt

Supplementary Table 2. Median repeat lengths observed among all polymorphic CGG repeat loci (*n*=5673) and the corresponding number of repeat loci that displayed each median repeat length.

See the attached txt file Supplementary Table 2.txt

Supplementary Table 3. Genes of interest. Genes associated with ID, AD, and CGG-repeats with their corresponding haploinsufficiency index, pLI score and cohort polymorphism rate (*n*=410).

See the attached txt file Supplementary Table 3.txt.

Supplementary Table 4. List of genes used genes used for routine screening for ID and related NDD (*n*=1295).

See the attached txt file Supplementary Table 4.txt.
